# An Area Partitioning and Subgraph Growing (APSG) Approach to the Conflation of Road Networks

**DOI:** 10.3390/s22041501

**Published:** 2022-02-15

**Authors:** Hoa-Hung Nguyen, Han-You Jeong

**Affiliations:** 1 Robotics Institute of Non-Destructive In-Line Inspection, Pusan National University, Busan 46241, Korea; nguyenhoahungit@gmail.com; 2 Department of Electrical Engineering, Pusan National University, Busan 46241, Korea

**Keywords:** road network conflation, area partitioning, subgraph growing, intelligent transportation systems

## Abstract

A road network represents a set of road objects in a geographic area and their interconnections, and it is an essential component of intelligent transportation systems (ITS) enabling emerging new applications such as dynamic route guidance, driving assistance systems, and autonomous driving. As the digitization of geospatial information becomes prevalent, a number of road networks with a wide variety of characteristics may coexist. In this paper, we present an *area partitioning and subgraph growing (APSG)* approach to the conflation of two road networks with a large difference in the level of details and representation rules. Our area partitioning (AP) scheme partitions the geographic area using the Network Voronoi Area Diagram (NVAD) of the low-detailed road network. Next, a subgraph of the high-detailed road network corresponding to a complex intersection is extracted and aggregated into a supernode so that high precision can be achieved via 1:1 road object matching. For the unmatched road objects due to missing road objects and different representation rules, we also propose a subgraph growing (SG) scheme that sequentially inserts a new road object while keeping the consistency of its connectivity to the matched road objects by the AP scheme. From the numerical results at Yeouido, Seoul, Korea, we show that our APSG scheme can achieve an outstanding matching performance in terms of the precision, recall, and F1-score.

## 1. Introduction

Geographic information systems (GIS) provide solutions for capturing, manipulating, analyzing, and visualizing the geospatial data for many application fields, such as transportation, agriculture, commerce, etc. [1,2]. Initially, government agencies have built authoritative GIS because the construction of geospatial information requires extensive and accurate surveys of the land [3,4]. Recently, as the digitization of geospatial information has recently become prevalent, some portal sites or mobile service providers have constructed proprietary GIS that combines authoritative GIS, aerial photos, mobile-mapping service (MMS), crowdsourcing data, etc. [5,6]. On the other hand, voluntary GIS, such as the openstreetmap (OSM), has been constructed by the participation of voluntary users carrying a GPS-enabled mobile terminal [7]. Currently, more than 7.8 million registered users all around the world contribute to the OSM [8].

A *road network* is a subset of GIS that focuses on road objects, attributes, and their interconnectivity. It is usually represented by a graph, where a node represents an intersection, an endpoint of a road, or a point of attribute change, whereas an edge represents a road segment connecting two nodes. The road network is an important component of many Intelligent Transportation System (ITS) applications. For example, turn-by-turn navigation establishes the shortest route connecting the origin and destination in the road network. In addition, the current traffic situation on the road segment is indexed by the corresponding identifier in the road network and then broadcast as public transportation data (PTD), which enables novel ITS applications, such as dynamic route guidance [9,10,11,12] and dynamic traffic management [13,14,15]. In a high-precision map for autonomous driving, each lane of a road can be represented in connection with the corresponding road segment of the road network [16].

Table 1 shows the characteristics of authoritative, proprietary, and voluntary road networks. First, the authoritative road network called a *node-link map (NLM)* is designed to support ITS applications in Korean major roads [4]. It provides the representation of a road object associated with its PTD attributes, such as average speed, road incidents, variable-message signs, and CCTV streamings [17]. Two major limitations of the NLM are the lack of software packages for ITS applications and the low-detailed representation of the road network. Second, the proprietary road network has good characteristics to support ITS services, but the access to its raw dataset and the ITS software packages is either very limited or impossible. The voluntary road network called the *OSM road network (ORN)* provides a detailed view of the road network based on the crowdsourcing GPS traces as well as a variety of open-source software packages: map editing tools (Potlatch 2 [18] and JOSM [19]), rendering tools (Mapnik [20] and the Tirex [21]), geocoding tools (Nominatim [22]), and especially routing tools (the open-source routing machine [23] and the Valhalla [24]). However, it has been reported that the quality of OSM objects obtained from crowdsourcing can be diverse in terms of accuracy, completeness, and consistency [25].

Taking into account the characteristics of road networks, we consider the *road network conflation (RNC)* between the authoritative and voluntary road networks, i.e., NLM and ORN, for emerging new ITS services. The RNC can be seen as a generalization of the road network matching (RNM) in [26,27,28,29,30,31,32,33,34,35,36,37,38,39,40]. Given two road networks, the RNM finds the association between a set of objects in one road network and another set in the other, where both sets represent the same road entity. Since the RNM is done without any modifications of input road networks, it cannot address the problem of missing road objects that can be found in the voluntary road networks [25]. The RNC relaxes this restriction by allowing to add road objects to one input road network. Since each road network has its own strengths and weaknesses, a successful RNC solution can enhance the strengths and compensate for the weaknesses. In particular, it can suggest a new direction to the emerging new ITS applications through the integration of NLM-indexed real-time transportation data with ORN software packages. The challenge of RNC is how to address the difference between two road networks, including level of details (LoD) [30,35,40], missing road objects [30,31,35], and representation rules.

In this paper, we present an *area partitioning and subgraph growing (APSG)* approach to the RNC that consists of two schemes: the area partitioning (AP) scheme for the RNM and the subgraph growing (SG) scheme for the unmatched NLM objects by the AP scheme. Our AP scheme exploits the network Voronoi area diagram (NVAD) in [41] to partition the map area into a set of regions centered on each node in the NLM graph. For each partitioned region, it extracts the ORN subgraph of a complex intersection and then aggregates it into an ORN supernode so that it can be associated with NLM node via 1:1 node matching. For the unmatched NLM subgraph due to missing road objects and different representation rules, we also propose the SG scheme that sequentially inserts an ORN road object corresponding to the unmatched NLM subgraph while keeping the consistency of its connectivity to the matched NLM subgraph by the AP scheme. The numerical results at Yeoui-do, Korea’s autonomous vehicle testing site, show that our APSG approach can achieve an outstanding RNC performance in terms of precision and recall. The contributions of this paper are summarized as follows:

- As far as we are aware, this is the first work to provide a formal definition of RNC that allows inserting new road objects into one road network only and presenting a novel APSG approach that achieves an outstanding matching performance;
- The proposed AP scheme can accurately cluster the nodes at a complex intersection not only by partitioning the map area using the NVAD but also extracting the precise subgraph that yields the maximum number of paths across the NVAD; and
- To address the problem of missing road objects and different representation rules, the proposed SG scheme inserts a new road object into the ORN subgraph so that it is as consistent as possible with the existing matchings by the AP scheme.

The remainder of this paper is organized as follows. Section 2 introduces the related works of the RNC. Section 3 describes the characteristics of two road networks and formulates the RNC problem. In Section 4, our AP scheme for the RNM is presented in detail. Section 5 presents the SG scheme for the unmatched NLM objects. The numerical results are discussed in Section 6, and finally, the conclusion of this paper is given in Section 7.

## 2. Related Work

Given two input road networks, RNM is the process of associating road objects and combining their attributes that represent the same road entity without any modifications of the input road networks. In the literature, numerous research efforts have focused on the RNM [27,28,29,30,31,32,33,34,35,36,37,38,39,40]. For a complete solution to RNM, it is necessary to comprehensively take into account the geometric and topological characteristics of all road objects in both input road networks. However, since it is difficult to reflect their global information, most of the existing approaches sequentially match a road object with its counterpart based on its local information. Depending on the type of matching road objects, the existing RNM approaches are classified into the node (or point) [26,27,28,29,30], path (or line) [31,32,33,34,36,37], and subgraph matching [38,39].

First, the node matching focuses on the matching between the points in the input road networks, such as intersections, traffic monitoring points, and the endpoints of overpass/underpass, bridges, and tunnels. The basic idea of node matching is to assess the proximity of the points to be matched as well as the similarity of their geometric and topological properties of incident edges. The seminal work in [26] presents an iterative scheme for RNM between the United States Geological Survey (USGS) and the Bureau of the Census: At each iteration, given a part of nodes already matched with their counterparts, the remaining nodes are relocated by the rubber–sheet transformation, and then, a new set of 1:1 node matchings is obtained again. In [27], the 1:1 node matching between two road networks with an order of scale difference exploits a few geometric dissimilarity measures, such as the Euclidean distance, nodal degree, and average orientation difference of incident edges. Given a matching of a node and its all neighbor nodes, paper [28] presents a round-trip walk scheme for evaluating the local topological consistency along the round-trip path across two road networks and the existing node matching. Although this paper also identifies the difficulties of 1:n and m:n node matchings, they are left as an open problem. By replacing the Euclidean distance of the DBSCAN clustering in [42] with the graph distance of road network, the authors in [30,40] present a node clustering scheme that aggregates the multiple nodes at a complex intersection into a single node. However, their clustering approach aggregating all intermediates nodes with an empirically determined stroke-length threshold may include too many nodes that do not belong to the complex intersection, which significantly degrades the overall matching performance. On the contrary, the proposed AP scheme can accurately cluster the nodes at the complex intersection not only by partitioning the whole map area based on the NVAD but also by extracting the precise subgraph that yields the maximum number of paths across the NVAD, which will be shown in Section 6.3.

Second, the path matching associates a path in one road network with another path in the other: Depending on the number of edges in each path, the path matching can be classified into 1:1, 1:n, m:1, and m:n edge matchings. A buffer-growing approach is proposed to address the most general m:n edge matching, where the merit function of potential matching pairs are computed by the mutual information of positions, angles, lengths, and forms within a two-hop distance, and the one with the highest mutual information is eventually selected as the matching pair [31]. An adaptive algorithm is proposed to determine the appropriate buffer size of the buffer-growing algorithm [32]: If the buffer size is too small, no candidate path can be found, and if the buffer size is too large, the computation complexity becomes high. However, the buffer-growing algorithm has two limitations: (1) to reduce the global errors between two input road networks, it requires an initial affine transformation using manually selected control points at the preprocessing step; and (2) to compute the mutual information, it also needs the statistical distribution of previously matched data from the same pair of input road networks, which is not usually available. A probabilistic relaxation scheme is also presented in [33], where it initializes the probability matrix based on the geometric dissimilarity of paths, iteratively updates the matching probabilities by evaluating the compatibility of neighbor candidate pairs, and selects the final 1:1 and 1:n matching pairs from the probability matrix. The probabilistic relaxation scheme in [34] improves the matching performance not only by considering both geometric and topological characteristics in the computation of probability matrix but also by inserting a virtual node in order to address the m:n matching pattern. To mitigate the user errors in the OSM crowdsourcing process, our APSG approach to the RNC problem also inserts a new node and edge into the ORN subgraph so that it can better match with the NLM.

Finally, the subgraph matching starts from an initial matching between the seed nodes, and the matched subgraph grows through a sequence of path and node matchings at each iteration. The semi-automated RNM in [38] consists of automated and interactive matching algorithms: The former includes the establishment of an initial matching for seed nodes and the expansion of the matching via cluster-based node/path matching algorithms, while the latter allows a human operator to manually correct the incorrect and improper initial matchings. On the other hand, the iterative matching algorithm in [39] initially performs the rubber–sheet transformation and topologically splits a path to maximize the number of 1:1 edge matchings. Then, starting from a subset of seed nodes, its combined edge and node matching algorithm gradually adds 1:1 matchings at the boundary of the existing matching set. Since the subgraph matching associates two *existing* road objects that represent the same road entity, its subgraph growing is determined by the similarity measure of their geometric and topological characteristics. The prime difference of our SG scheme is that *new road objects* are sequentially inserted into the subgraph of one road network to address the problem of missing road objects and different representation rules. In this process, the order of inserted road objects is carefully determined so that the resulting subgraph is as consistent as possible with the existing matchings by the AP scheme.

## 3. Input Road Networks and Problem Specification

In this section, we describe the characteristics of two road networks, i.e., NLM and ORN, and then formulate the RNC problem.

### 3.1. Node-Link Map

The Korean government has initiated the national GIS project in 1995 and completed the construction of the geospatial database in 2009 [43]. The NLM is the road network of this database that represents major road objects in Korea [4]. It also provides a unified identifier (ID) hierarchy to its road entity. In order to efficiently exchange the ITS information, the Korean law enforces that all ITS applications must use the NLM ID hierarchy to exchange road and traffic information [17].

**Figure 1:**
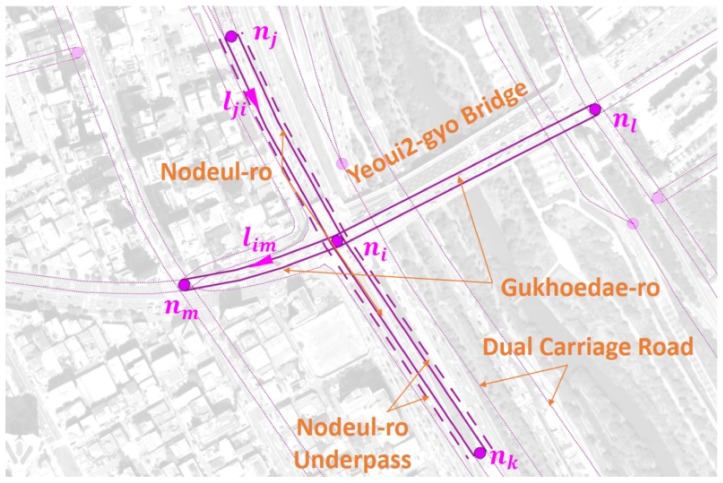
NLM graph representation around Yeoui2-gyo intersection.

Figure 1 shows an NLM graph representation of the Yeoui2-gyo intersection, Yeoui-do, Seoul, Korea overlaid on top of the aerial view, where Gukhoe-daero (east-west road) and the access ramps of Nodeul-ro (north-south underpass) are interconnected. The NLM graph is a *directed* graph $G_{N}=\left(N,L\right)$, where N is the set of nodes representing the points at which the road characteristics are changed and L is the set of links connecting two nodes. The A node can be an intersection ($n_{i},n_{l}$, and $n_{m}$), traffic monitoring point, administrative boundary, and the endpoints of the road, overpass, and underpass ($n_{j}$ and $n_{k}$). A single NLM node $n_{i}\in N$ is used to represent a complex intersection (Yeoui2-gyo) without a detailed view of the internal road network. We define the subgraph $G_{N}\left(n_{i}\right)=\left(N\left(n_{i}\right),L\left(n_{i}\right)\right)$, which consists of an NLM node $n_{i}$, its directly connected links (pink solid links in $G_{N}$), and the neighbor NLM nodes ($n_{j},n_{k},n_{l}$, and $n_{m}$). An NLM node is placed at the crosspoint of two roads, where a road consists of two parallel links, each of which represents a unidirectional road segment. In a dual carriage road, it is placed at the endpoint of two NLM links.

The NLM link set L consists of unidirectional links, where its element $l_{xy}=\left(n_{x},n_{y}\right)$ is an ordered pair of nodes directed from $n_{x}$ to $n_{y}$. The geometric shape of a link is approximated by a sequence of concatenated line segments. For example, unidirectional links $l_{ji}$ and $l_{im}$ are shown by pink solid lines with triangular marks for their directions. The underpass and overpass links are also placed in parallel with the main road segment with additional spacing between them. In this paper, we represent each NLM underpass/overpass by the pink dashed line, as shown in Figure 1. Each link has a set of attributes, such as *link_id*, *f_node*, *t_node*, *road_rank*, *road_type*, *connect*, *road_use*, etc., where the *road_rank* attribute represents the class of road segment as shown in Table 2, *road_type* specifies the type of road, such as overpass, underpass, bridge, tunnel, etc., *connect* specifies the type of ramps depending on the *road_rank* attribute, and *f_node* and *t_node* represent the start and end node indexes of the NLM link, respectively.

### 3.2. OpenStreetMap Road Network (ORN)

The ORN is a subset of OSM objects with the *highway* tag, where a tag is an ordered pair of (key, value) identifying the attribute of a road object. Table 3 shows the *highway* tag of way, which is classified into a few groups. In each group, the tag values are ordered from the most important to the least important. The main focus of this paper is on the *road* and *link road* groups, where the former is a way of representing a road while the latter is a way of connecting two roads in a complex intersection. We initially prune all ORN objects in *special roads*, *paths*, *sidewalks*, and *cycleways* groups that do not correspond to the NLM objects. This pruning process removes approximately 20% of unnecessary road objects from the original ORN. Furthermore, we also remove the subgraphs for underpass/overpass in both road networks because they can be easily matched via their attributes, such as NLM *road_type* and ORN *tunnel* and *bridge* tags (In some figures, we still illustrate the ORN underpass/overpass with a green dashed line for the clarity of expression).

**Figure 2:**
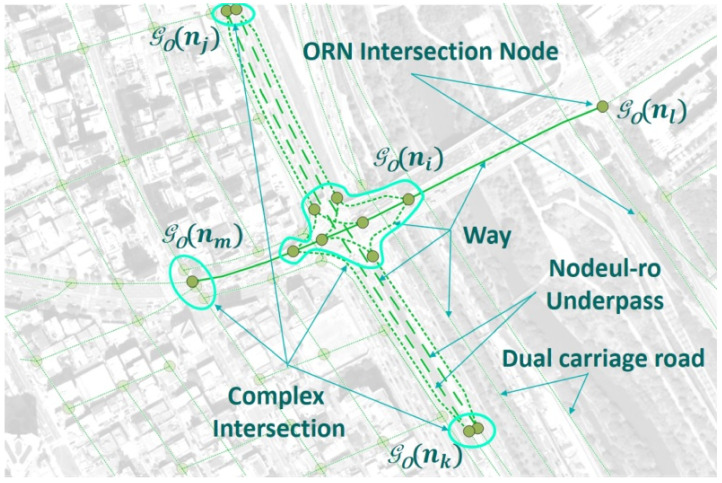
ORN graph representation around Yeoui2-gyo intersection.

Figure 2 shows the ORN graph representation, which can be modeled by an *undirected* graph $G_{O}=\left(V,ℰ\right)$. Contrary to the NLM graph $G_{N}$, the ORN graph $G_{O}$ is designed to reflect the detailed road network at a complex intersection. This feature makes the ORN more suitable for ITS applications, such as navigation and autonomous driving.

In $G_{O}$, an ORN node v∈V is connected to at least three neighbor ORN nodes. In the RNC, NLM node *n* is associated with ORN subgraph $G_{O}\left(n\right)$, where the ORN subgraph can be a single ORN intersection node, e.g., $G_{O}\left(n_{l}\right)$, disconnected subgraphs, e.g., $G_{O}\left(n_{j}\right)$ and $G_{O}\left(n_{k}\right)$, or a connected subgraph, e.g., $G_{O}\left(n_{i}\right)$ and $G_{O}\left(n_{m}\right)$, in Figure 2. If an intersection consists of a single ORN intersection node, it is called a simple intersection; otherwise, it is called a complex intersection.

The atomic unit for representing an ORN road is a way w∈W, which may span multiple ORN nodes [7]. If way *w* includes more than two ORN nodes, it is decomposed into consecutive ORN edges e∈ℰ so that each edge connects two ORN nodes only. In Figure 2, the Gukhoe-daero in the ORN subgraph $G_{O}\left(n_{i}\right)$ consists of edges with the *road* tag group only, as shown in solid green lines, whereas all remaining edges in $G_{O}\left(n_{i}\right)$ belong to the *link road* tag group, as represented by dotted green lines. On the other hand, all intersecting edges at a simple intersection, such as $G_{O}\left(n_{l}\right)$, belong to the *road* tag group. In the case of a dual carriage road, a distinct edge is used for each ORN edge, whose direction is specified to the *direction* tag.

### 3.3. Problem Specification

**Figure 3:**
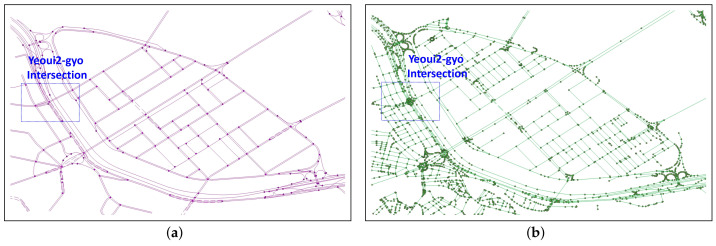
NLM and ORN graph representation of Yeoui-do roads: (**a**) NLM graph and (**b**) (pruned) ORN graph.

Figure 3 shows the NLM and ORN graph representation of Yeoui-do roads, which are given as the input of our RNC problem. The NLM in Figure 3a is a low-detailed road network consisting of major public roads only, while the ORN in Figure 3b has a much more detailed representation of the road network. Given NLM $G_{N}$ and ORN $G_{O}$, the RNC problem is an association problem that finds the ORN subgraph corresponding to each NLM object while allowing to add new road objects to the ORN.

**Figure 4:**
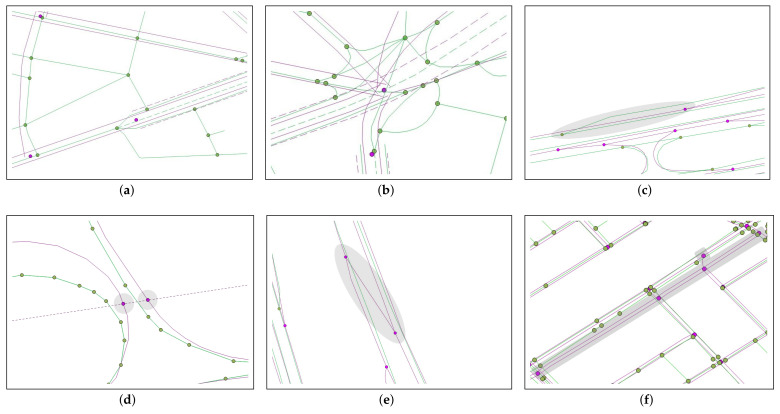
Examples of the representational dissimilarities between NLM and ORN: (**a**) Number of road objects, (**b**) LoD difference at complex intersection, (**c**) different rules to represent merging lane, (**d**) no corresponding ORN nodes, (**e**) no corresponding ORN edge, and (**f**) no corresponding ORN subgraph.

Since each road network has its own rules for representing its road objects, there are several differences in representing road objects between two road networks, as shown in Figure 4: Figure 4a shows different numbers of road objects, where the ORN shows both major and minor roads in a geographical area, while the NLM displays major public roads only. Figure 4b illustrates different LoDs at a complex intersection, where the ORN illustrates all detailed connectivity at the intersection, whereas the NLM aggregates them into a single NLM node. Figure 4c reveals two different rules to represent a merging lane, where it is a part of the mainline road in the NLM, while it is a part of the on-ramp with the *trunk_link* tag in the ORN. Figure 4d shows two NLM nodes that do not have the corresponding ORN subgraphs at the crosspoints of the administrative boundary. Figure 4e illustrates an NLM link without the corresponding ORN object due to its omission during the crowdsourcing process of OSM. Figure 4f also shows an NLM subgraph that does not have the corresponding ORN subgraph due to the OSM crowdsourcing errors.

To summarize, a comprehensive solution to the RNC problem needs to address the fundamental issues of these representational differences, as follows:

1. To identify the ORN subgraph of a complex intersection in order to alleviate the LoD difference between two road networks;
2. To find a reliable methodology to cope with the differences in the representation of merging lane and administrative boundary; and
3. To create a new ORN subgraph corresponding to the unmatched NLM subgraph while keeping the consistency of its connectivity to the matched NLM subgraph.

## 4. Area Partitioning for LoD Difference at a Complex Intersection

For a given NLM node $n_{i}$ with NLM subgraph $G_{N}\left(n_{i}\right)$ and ORN graph $G_{O}=\left(V,ℰ\right)$, the challenging task is to accurately extract ORN subgraph $G_{T}\left(n_{i}\right)$ against a wide variety of intersection topology, as shown in Figure 4b. An inaccurate ORN subgraph incurs an incorrect matching, which in turn influences the accuracy of another matching. This propagation eventually results in severe degradation of RNC performance.

Our AP scheme first computes the region of the map dedicated to NLM node $n_{i}$ in which the corresponding ORN subgraph may exist. Then, it extracts ORN subgraph $G_{O}^{*}\left(n_{i}\right)$ along the path connecting each pair of entering and exiting points across the region boundary, taking into account the turning information and geometry of intersection. Finally, ORN subgraph $G_{O}^{*}\left(n_{i}\right)$ is replaced by an ORN supernode $v_{i}^{*}$ so that it can be matched with NLM node $n_{i}$ via 1:1 node matching.

### 4.1. Network Voronoi Area Diagram (NVAD) for Partitioning Map Area

**Figure 5:**
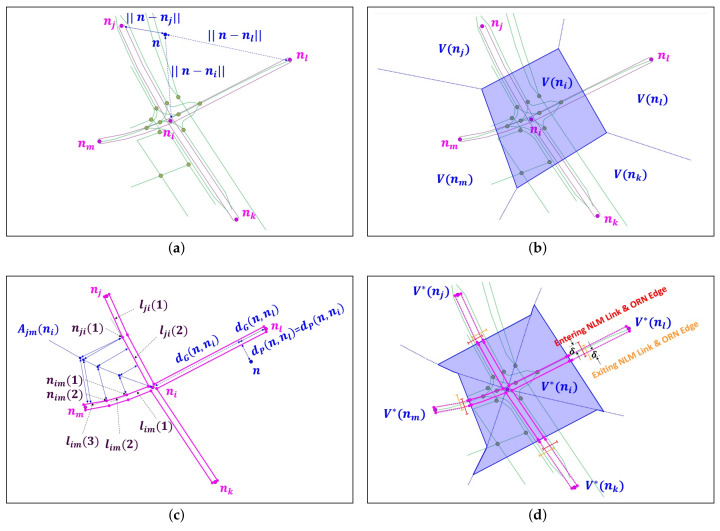
VD and NVAD to partition map area $A(n_{i})$ around NLM node $n_{i}$: (**a**) Euclidean distance of Voronoi diagram, (**b**) Voronoi cell $V(n_{i})$ for NLM node $n_{i}$, (**c**) distance metrics and projection boundary of NVAD, and (**d**) NVAD $V^{*}\left(n_{i}\right)$ for NLM node $n_{i}$.

Given NLM subgraph $G_{N}\left(n_{i}\right)$ and the corresponding map area $A(n_{i})$ around $n_{i}$, the first task of our AP scheme is to partition this area into regions, where each region is centered at an intersection in $N(n_{i})$. If we focus on NLM node $N(n_{i})$ without considering NLM subgraph $G_{N}\left(n_{i}\right)$, a simple method to partition the map area $A(n_{i})$ based on the Euclidean distance is called the *Voronoi diagram (VD)* [41]. In the VD, a point $n\in A(n_{i})$ is associated with the region of the closest intersection $n_{x}$, called the Voronoi cell $V(n_{x})$, in terms of the Euclidean distance metric:

(1) $$V\left(n_{x}\right)=\{n|\;∥n-n_{x}∥\leq ∥n-n_{y}∥\;\forall y\neq x,\;n_{x},n_{y}\in N\left(n_{i}\right)\},$$

where $N\left(n_{i}\right)=\left\{n_{i},n_{j},n_{k},n_{l},n_{m}\right\}$ for NLM graph $G_{N}\left(n_{i}\right)$ in Figure 5a. Given an ORN node $n\in V(n_{j})$ in map area $A(n_{i})$, the Euclidean distances from the three closest NLM nodes are shown in Figure 5a. For two NLM nodes $n_{x}$ and $n_{y}$ ($n_{y}\in N\left(n_{i}\right)\backslash \left\{n_{x}\right\}$), the boundary of Voronoi cells becomes a hyperplane that is equidistant from both NLM nodes. Finally, Voronoi cell $V(n_{x})$ is constructed by intersecting all half-spaces in which NLM node $n_{x}$ is located. For example, Voronoi cell $V(n_{i})$ is illustrated with the blue transparent quadrilateral in Figure 5b.

However, given the geometry of NLM subgraph $G_{N}\left(n_{i}\right)$, the Euclidean norm is no longer a valid measure to evaluate the distance between point $n\in A(n_{i})$ and the set of NLM nodes in $N(n_{i})$, because it does not account for the distance between two points in an arbitrary road network $G_{N}$. The network Voronoi node and link diagrams in [41,44] can be used for the measure of graph distance, but they are limited to the points on NLM subgraph $G_{N}\left(n_{i}\right)$ only and are not applicable for an arbitrary point in area $A(n_{i})$. To address this problem, our AP scheme adopts the *Network Voronoi Area Diagram (NVAD)* whose measure reflects two distance factors [41]: First, if point *n* is on subgraph $G_{N}\left(n_{i}\right)$, the distance should be the length of shortest path to NLM node $n_{x}\in N\left(n_{i}\right)$ in $G_{N}\left(n_{i}\right)$, which is called the graph distance $d_{G}\left(n,n_{x}\right)$. If point *n* lies in $A\left(n_{i}\right)\backslash G_{N}\left(n_{i}\right)$, the measure should also consider the projection distance $d_{P}\left(n,n_{x}\right)$ to the closest NLM link of subgraph $G_{N}\left(n_{i}\right)$. Figure 5c shows these distances between point *n* and the two closest intersections, $n_{i}$ and $n_{l}$. Consequently, the distance metric of NVAD is defined as the sum of these two distance components, i.e.,

(2) $$∥n-n_{x}∥=d_{G}\left(n,n_{x}\right)+d_{P}\left(n,n_{x}\right).$$

To determine the NLM link onto which a given point *n* is projected, we choose an example of map area $A_{jm}\left(n_{i}\right)$ surrounded by unidirectional NLM links $l_{ji}$ and $l_{im}$ in Figure 5c, where the former (latter) consists of two (three) line segments. The *k*-th line segment and vertex of NLM link $l_{ji}$ are denoted by $l_{ji}\left(k\right)$ and $n_{ji}\left(k\right)$, respectively, where $n_{ji}\left(0\right)=n_{j}$ and $n_{ji}\left(2\right)=n_{i}$. Our approach draws the equiangle boundary starting from the center of intersection $n_{i}$ until its projection approaches the endpoint of the shorter line segment $n_{im}\left(1\right)$. Notice that any points on this projection boundary are equidistant from both NLM links $l_{ji}$ and $l_{im}$. In Appendix A, we demonstrate that the projection boundary curve becomes a concatenation of linear or parabolic segments. Figure 5c shows the resulting blue dotted projection boundary of map area $A_{jm}\left(n_{i}\right)$.

Figure 5d shows all projection boundaries that partition map area $A(n_{i})$ into four projection areas, each of which has a pair of NLM links between $n_{i}$ and its neighbor NLM node. At the middle point of these links, we draw a perpendicular line that bisects the projection area. Then, NVAD cell $V^{*}\left(n_{i}\right)$ is determined by the union of the bisected map area in which NLM node $n_{i}$ is located, as shown by the blue transparent polygon in Figure 5d. For each NLM link *l* passing through the NVAD cell boundary, we finally build a list of candidate ORN edges ${ℰ}_{l}=\left\{e_{l}\left(1\right),e_{l}\left(2\right),\cdots \right\}$ of the same direction whose distance along the boundary line is less than threshold δ. For example, in Figure 5d, NLM links $l_{ji}$ and $l_{ik}$ have two ORN edges in their lists, while all remaining NLM links have only one ORN edge. In the next section, the candidate ORN edges will be examined to be the correspondent of an NLM link.

### 4.2. Extraction of Candidate ORN Subgraph

**Figure 6:**
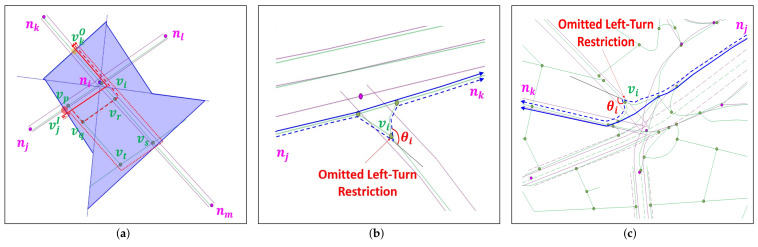
Candidate ORN paths between the ORN edges closest to $l_{ji}$ and closest to $l_{ik}$ at the boundary of NVAD cell $V^{*}\left(n_{i}\right)$ of (**a**) simple intersection, (**b**) exceptional case I, and (**c**) exceptional case II.

Given NVAD cell $V^{*}\left(n_{i}\right)$, the allowable turn information at NLM node $n_{i}$, and set ${ℰ}_{l}$ of candidate ORN edges for NLM link *l*, the objective of this section is to extract the corresponding ORN subgraph $G_{O}^{*}\left(n_{i}\right)$ in $V^{*}\left(n_{i}\right)$ that corresponds to NLM node $n_{i}$. Our key observation is that an intersection allows at most one path for each pair of roads, where one enters to and the other exits from NVAD cell $V^{*}\left(n_{i}\right)$. Starting with the null ORN subgraph having no ORN node and edge, the basic idea of our approach is to sequentially insert an ORN path passing through the intersection along which the turn restriction is satisfied at each pair of consecutive ORN edges. Without loss of generality, we focus on the construction of ORN path $p_{jk}$, as shown in Figure 6.

Figure 6a shows an example of a simple intersection, where NLM node $n_{i}$ connects a two-way road ($l_{ki}$ and $l_{ik}$) and three one-way roads ($l_{ji}$, $l_{il}$, and $l_{mi}$). Due to the LoD difference, the ORN subgraph in $V^{*}\left(n_{i}\right)$ consists of two components: (1) the true ORN subgraph almost overlapped with NLM subgraph $G_{N}\left(n_{i}\right)$, and (2) the remaining ORN subgraph representing a minor road network around intersection $n_{i}$. Denoting by $v_{j}^{I}$ and $v_{k}^{O}$ the crosspoints of the entering and exiting ORN edges at the boundary of NVAD cell $V^{*}\left(n_{i}\right)$, respectively, there are three candidate paths in Figure 6a: $p_{jk}\left(1\right)=v_{j}^{I}\to v_{i}\to v_{k}^{O}$ (red solid path), $p_{jk}\left(2\right)=v_{j}^{I}\to v_{p}\to v_{q}\to v_{r}\to v_{k}^{O}$ (red dashed path), and $p_{jk}\left(3\right)=v_{j}^{I}\to v_{p}\to v_{t}\to v_{s}\to v_{k}^{O}$ (red dotted path). Among these paths, our *candidate ORN subgraph extraction (COSE)* scheme chooses the path that has smallest sum of turning angles regardless of its direction. For example, path $p_{jk}\left(1\right)$ has the smallest total turning angle since it makes only one left-turn at $v_{i}$ compared to three turns in the other two paths.

Although ORN subgraph $G_{O}^{*}\left(n_{i}\right)$ is much more complex than NLM subgraph $G_{N}\left(n_{i}\right)$ around a complex intersection, it is surprising that our key observation is valid for all complex intersections in Yeoui-do except for the blue dashed paths in Figure 6a,b. They are evidently originated from the crowdsourcing error that omits a left-turn restriction at ORN node $v_{i}$ by the participating users, and eventually, they turn out to be invalid paths. Unfortunately, these human errors are inevitable in the crowdsourcing-based ORN. To exclude these exceptional paths from ORN subgraph $G_{O}^{*}\left(n_{i}\right)$, we exploit the second key observation that *the geometry of connecting roads in a complex intersection is designed in a way that the curvature changes linearly with the curve length*, which is known as the *clothoids*. Based on this observation, the COSE scheme discards a path if it has two consecutive edges and the angles between them abruptly change, e.g., the blue dashed path at node $v_{i}$ in Figure 6a,b.

**Figure 7:**
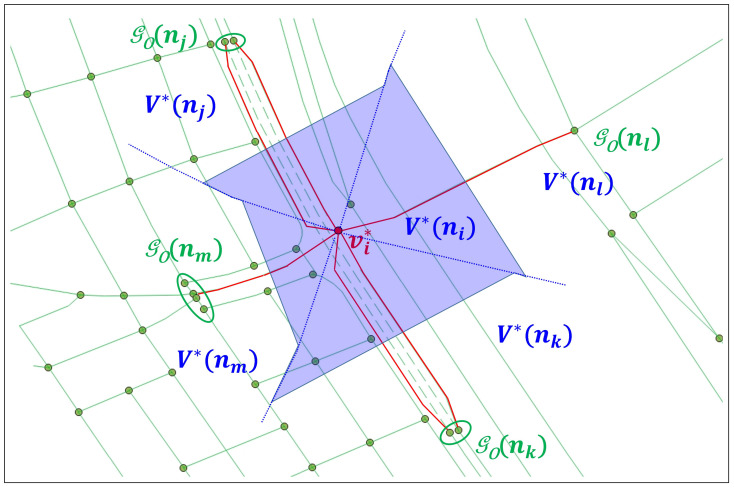
ORN supernode $v_{i}^{*}$ replacing the final ORN subgraph $G_{O}^{*}\left(n_{i}\right)$.

The final step of the COSE scheme is the derivation of ORN subgraph $G_{O}^{*}\left(n_{i}\right)$ for NVAD node $n_{i}$. It first calculates the number of allowable ORN paths for each ingress-egress pair of ORN edges at the boundary of NVAD cell $V^{*}\left(n_{i}\right)$. Then, it chooses the optimal ORN subgraph $G_{O}^{*}\left(n_{i}\right)=\left(V^{*}\left(n_{i}\right),\;{ℰ}^{*}\left(n_{i}\right)\right)$ that yields the largest number of allowable ORN paths. Finally, it extracts all ORN nodes from $V^{*}\left(n_{i}\right)$. If there is more than one ORN node in $V^{*}\left(n_{i}\right)$, our AP approach replaces them with ORN supernode $v_{i}^{*}$ located at the center of them, as shown in Figure 7. By this replacement, the node matching becomes a simple 1:1 matching between NLM node $n_{i}$ and ORN supernode $v_{i}^{*}$.

### 4.3. Classification of RNM Result

**Figure 8:**
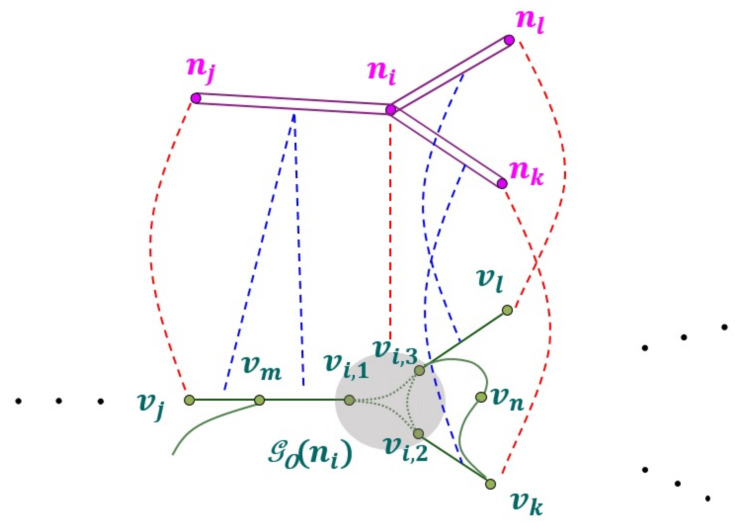
Example of RNM results.

Figure 8 shows the matching results between NLM subgraph $G_{N}\left(n_{i}\right)$ and ORN graph $G_{O}$. Depending on which road object belongs to ORN subgraph $G_{O}^{*}\left(n_{i}\right)$, both node and edge matching results can be one of the following four matching types: *correct match (CM)*, *incorrect match (IM)*, *partial match (PM)*, and *missing match (MM)*. For each NLM node, the node matching result can be determined as follows:

- The red dashed lines in Figure 8 represent the CM between NLM and ORN nodes, where the sets of true ORN nodes for NLM nodes $n_{i},\;n_{j},\;n_{k},$ and $n_{l}$ are denoted by $V_{T}\left(n_{i}\right)=\left\{v_{i,1},v_{i,2},v_{i,3}\right\},$$V_{T}\left(n_{j}\right)=v_{j},$$V_{T}\left(n_{k}\right)=v_{k},$ and $V_{T}\left(n_{l}\right)=v_{l}$, respectively;
- A node matching becomes MM if its set of ORN nodes is empty, i.e., $V^{*}\left(\;\cdot \;\right)=\phi$;
- A node matching becomes IM if its set of ORN nodes is *disjoint* with the set of true ORN nodes, i.e., $V^{*}\left(\;\cdot \;\right)\cap V_{T}\left(\;\cdot \;\right)=\phi$; and
- A node matching becomes PM if its set of ORN nodes satisfies two conditions $V^{*}\left(\;\cdot \;\right)\cap V_{T}\left(\;\cdot \;\right)\neq \phi$ and $V^{*}\left(\;\cdot \;\right)\neq V_{T}\left(\;\cdot \;\right)$.

At the boundary of two adjacent NVAD cells $V^{*}\left(n_{i}\right)$ and $V^{*}\left(n_{j}\right)$, the COSE scheme also yields a solution to the edge matching between NLM link *l* and two ORN edges: one from set ${ℰ}_{l}\cap {ℰ}^{*}\left(n_{i}\right)$ in area $A(n_{i})$ and the other from ${ℰ}_{l}\cap {ℰ}^{*}\left(n_{j}\right)$ in area $A(n_{j})$, respectively. Similarly, the type of edge matching result is determined as follows:

- The blue dashed lines in Figure 8 represent the CM between NLM link and ORN edges, where the sets of true ORN edges for NLM link $l_{ij},\;l_{ik},$ and $l_{il}$ are denoted by ${ℰ}_{T}\left(l_{ij}\right)=\left\{\left(v_{i,1},\;v_{m}\right),\left(v_{m},\;v_{j}\right)\right\},$${ℰ}_{T}\left(l_{ik}\right)=\left(v_{i,2},\;v_{k}\right),$ and ${ℰ}_{T}\left(l_{il}\right)=\left(v_{i,3},\;v_{l}\right),$ respectively;
- An edge matching becomes MM if its set of ORN edges is empty, i.e., ${ℰ}^{*}\left(\;\cdot \;\right)=\phi$;
- An edge matching becomes IM if its set of ORN edges is *disjoint* with the set of true ORN edges, i.e., ${ℰ}^{*}\left(\;\cdot \;\right)\cap {ℰ}_{T}\left(\;\cdot \;\right)=\phi$; and
- An edge matching becomes PM if its set of ORN edges satisfies two conditions ${ℰ}^{*}\left(\;\cdot \;\right)\cap {ℰ}_{T}\left(\;\cdot \;\right)\neq \phi$ and ${ℰ}^{*}\left(\;\cdot \;\right)\neq {ℰ}_{T}\left(\;\cdot \;\right)$.

Finally, we partition NLM graph $G_{N}$ into the matched and unmatched NLM subgraphs $G_{N}^{*}=\left(N^{*},L^{*}\right)$ and ${\bar{G}}_{N}=\left(\bar{N},\bar{L}\right)$, where the former includes all NLM road objects of CM, IM, and PM types, while the latter has those in MM type only.

## 5. ORN Subgraph Growing for Unmatched NLM Subgraph

The unmatched NLM subgraph is mainly originated from missing ORN objects in the OSM crowdsourcing process or the differences in representation rule. In general, a connected subgraph of unmatched NLM graph ${\bar{G}}_{N}$ can be either NLM node ${\bar{n}}_{i}$, NLM link ${\bar{l}}_{ij}$, or NLM component ${\bar{C}}_{N}$ consisting of at least two NLM road objects. First, we present two schemes for unmatched single NLM node due to the differences in representation rule: the NVAD cell expansion (NCE) scheme for a merging lane in Figure 4c and the NLM node projection onto the ORN edge (NPOE) scheme for administrative boundaries in Figure 4d. Second, we present the ORN edge insertion (OEI) scheme for the unmatched single NLM link in Figure 4e. Finally, we present the sequential ORN subgraph growing (SOSG) scheme for the unmatched NLM component in Figure 4f. Finally, we also address the internal structure design of new ORN nodes by the SG scheme.

### 5.1. Schemes for Unmatched Single NLM Node

**Figure 9:**
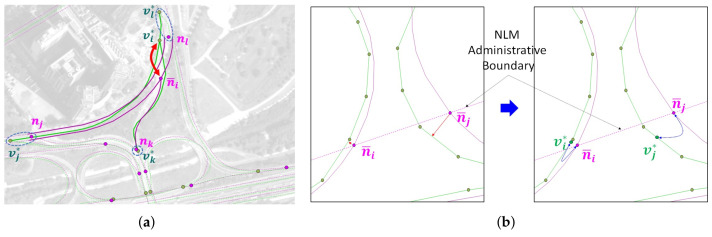
Examples of unmatched single NLM node ${\bar{n}}_{i}$ in (**a**) merging lane and (**b**) administrative boundary.

We present two schemes to address the difference in representation rule: the NCE scheme for the merging lane and the NPOE scheme for the administrative boundary. This difference results in an isolated NLM node, as shown in Figure 9.

#### 5.1.1. NCE Scheme for Merging Lane

Figure 9a shows a typical example of different rules for representing a merging lane, where it is a part of the mainline road in NLM while it is a part of the on-ramp in ORN. As a result, the ORN edge connecting $v_{k}^{*}$ and $v_{i}^{*}$ is longer than the corresponding NLM link $l_{ki}$. This rule difference results in unmatched single NLM node ${\bar{n}}_{i}$ with $|L({\bar{n}}_{i})|\geq 3$, because its corresponding ORN node $v_{i}^{*}$ is located outside its NVAD cell $V^{*}\left({\bar{n}}_{i}\right)$.

To address this problem, we present the NCE scheme as follows: It first expands its NVAD cell $V^{*}\left({\bar{n}}_{i}\right)$ through the union of all NVAD cells in map area $A({\bar{n}}_{i})$, i.e., $\cup V^{*}\left(n_{x}\right)$ for each $n_{x}\in N\left(n_{i}\right)$. Next, the COSE scheme in Section 4.2 is used to extract the corresponding ORN node $v_{i}^{*}$ from all possible ORN paths, e.g., paths $v_{j}^{*}\to v_{l}^{*}$ and $v_{k}^{*}\to v_{l}^{*}$ in Figure 9a.

#### 5.1.2. NPOE Scheme for Administrative Boundary

Figure 9b shows an example of different rules for indicating a road crossing an administrative boundary: Two nodes ${\bar{n}}_{i}$ and ${\bar{n}}_{j}$ are created to represent the administrative boundary in the NLM links, while no corresponding ORN node exists in NVAD cells $V^{*}\left({\bar{n}}_{i}\right)$ and $V^{*}\left({\bar{n}}_{j}\right)$, respectively. To address this problem, we propose the NPOE scheme that projects the unmatched NLM nodes ${\bar{n}}_{i}$ and ${\bar{n}}_{j}$ onto the ORN subgraphs $G_{O}\left({\bar{n}}_{i}\right)$ and $G_{O}\left({\bar{n}}_{j}\right)$ obtained from the COSE scheme, respectively. For example, Figure 9b shows two ORN nodes $v_{i}^{*}$ and $v_{j}^{*}$ that are matched with unmatched NLM nodes ${\bar{n}}_{i}$ and ${\bar{n}}_{j}$, respectively. If the unmatched NLM node is on dual carriage roads, the NPOE scheme collapses the projected ORN nodes into an ORN node located at the middle of them.

### 5.2. OEI Scheme for Missing ORN Edge

**Figure 10:**
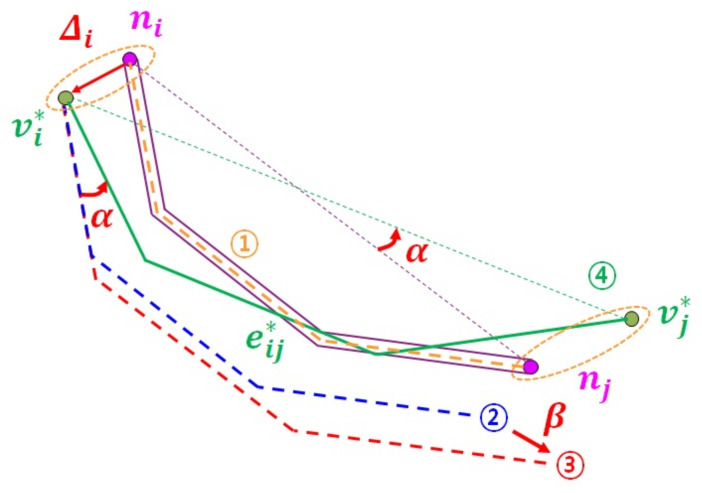
Example of OEI scheme for correspondent-missing NLM link ${\bar{l}}_{ij}$.

Figure 10 shows an example of OEI scheme to address the problem that there is no ORN edge corresponding to NLM link ${\bar{l}}_{ij}$. In this example, both endpoints $n_{i}$ and $n_{j}$ of NLM link ${\bar{l}}_{ij}$ are matched with ORN nodes $v_{i}^{*}$ and $v_{j}^{*}$ via the AP scheme, respectively. However, the ORN edge connecting these ORN nodes is missing possibly due to user errors in the OSM crowdsourcing process. The goal of this section is to insert an ORN edge $e_{ij}^{*}$ that corresponds to NLM link ${\bar{l}}_{ij}$. To this aim, our OEI scheme considers three factors: (1) the displacement $\Delta_{i}$ between NLM node $n_{i}$ and ORN node $v_{i}^{*}$, (2) the angle difference α between NLM line segment $\left(n_{i},\;n_{j}\right)$ and ORN line segment $\left(v_{i}^{*},\;v_{j}^{*}\right)$, and (3) the length ratio β of ORN line segment $\left(v_{i}^{*},\;v_{j}^{*}\right)$ to NLM line segment $\left(n_{i},\;n_{j}\right)$, where

(3) $$\beta =\frac{∥v_{i}^{*}-v_{j}^{*}∥}{∥n_{i}-n_{j}∥}.$$

The OEI scheme first computes an orange dashed link between NLM nodes $n_{i}$ and $n_{j}$, which is equally distant from both NLM links ${\bar{l}}_{ij}$ and ${\bar{l}}_{ji}$. Next, it obtains a blue dashed link by shifting the orange dashed link by $\Delta_{i}$ so that it can start from ORN node $v_{i}^{*}$. Then, it computes a red dashed link by multiplying the scaling factor β to the blue dashed line. Finally, ORN edge $e_{ij}^{*}$ in Figure 10 is obtained by rotating the red dashed link by angle α around ORN node $v_{i}^{*}$.

### 5.3. SOSG Scheme for Unmatched NLM Component

**Figure 11:**
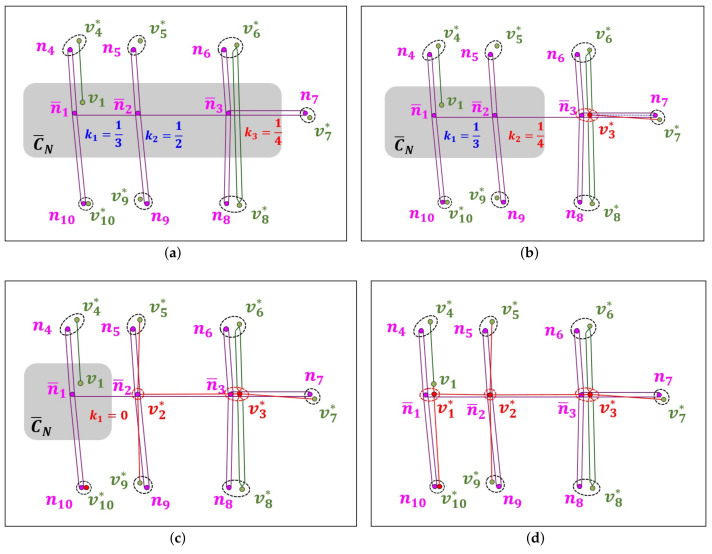
Example of SOSC scheme for unmatched NLM component ${\bar{C}}_{N}$: (**a**) Initial unmatched NLM component ${\bar{C}}_{N}$, (**b**) after the extraction of ${\bar{n}}_{3}$ from *Q*, (**c**) after the extraction of ${\bar{n}}_{2}$ from *Q*, and (**d**) the ORN subgraph $G_{O}\left({\bar{C}}_{N}\right)$ for ${\bar{C}}_{N}$.

During the OSM crowdsourcing process, the ORN subgraph $G_{O}\left({\bar{C}}_{N}\right)$ corresponding to unmatched NLM component ${\bar{C}}_{N}$ may not exist due to the misinterpretation of the road network (see the example in Figure 4f). Figure 11a shows an example of NLM component ${\bar{C}}_{N}$ consisting of three unmatched NLM nodes (${\bar{n}}_{1}$, ${\bar{n}}_{2}$, and ${\bar{n}}_{3}$), and 16 unmatched NLM links: Two unmatched NLM links connect two unmatched NLM nodes in ${\bar{C}}_{N}$, while 14 unmatched NLM links pass through the boundary of ${\bar{C}}_{N}$. The objective of our SOSC scheme is to construct a simple ORN subgraph $G_{O}\left({\bar{C}}_{N}\right)$ that corresponds to NLM component ${\bar{C}}_{N}$.

The basic idea of the SOSC scheme is to sequentially examine an unmatched NLM node in ${\bar{C}}_{N}$, and for each unmatched NLM node, to construct the corresponding ORN subgraph using both OEI and NPOE schemes. It maintains priority queue *Q* that determines the order of unmatched NLM nodes sequentially extracted from ${\bar{C}}_{N}$. To better associate with the neighbor NLM nodes in matched NLM subgraph $G_{N}^{*}$, the key $k_{i}$ of unmatched NLM node ${\bar{n}}_{i}$ in priority queue *Q* is defined as the ratio of unmatched neighbor NLM nodes to all neighbor NLM nodes $N\left({\bar{n}}_{i}\right)\backslash \left\{{\bar{n}}_{i}\right\}$. Since the three key values are $k_{1}=\frac{1}{3}$, $k_{2}=\frac{1}{2}$, and $k_{3}=\frac{1}{4}$ in Figure 11a, NLM node ${\bar{n}}_{3}$ is first extracted from *Q*.

For unmatched NLM node ${\bar{n}}_{3}$ extracted from *Q*, it first investigates the existence of an ORN edge corresponding to NLM links $l_{3j}$ and/or $l_{j3}$, where NLM node $n_{j}$ belongs to the set of matched neighbor NLM nodes $N\left({\bar{n}}_{3}\right)\cap N^{*}$. Figure 11a shows a dual carriage edge between two neighbor ORN nodes $v_{6}^{*}$ and $v_{8}^{*}$. In this case, it uses the NPOE scheme to insert ORN node $v_{3}^{*}$ to the center of two projection points onto ORN edges $\left(v_{6}^{*},\;v_{8}^{*}\right)$ and $\left(v_{8}^{*},\;v_{6}^{*}\right)$ in Figure 11b. Once ORN node $v_{3}^{*}$ is created, the OEI scheme is used to insert a new ORN edge $e_{37}^{*}$. Then, unmatched NLM node ${\bar{n}}_{3}$ and new NLM links $l_{36},l_{63},l_{37},l_{38},$ and $l_{83}$ that have their corresponding ORN edges are removed from NLM component ${\bar{C}}_{N}$ and then inserted to matched NLM subgraph $G_{N}^{*}$, which reduces the key value of unmatched NLM node ${\bar{n}}_{2}$ to $k_{2}=\frac{1}{4}$, as shown in Figure 11b.

Next, unmatched NLM node ${\bar{n}}_{2}$ extracted from *Q* is examined to find an existing ORN edge corresponding to NLM links $l_{23},l_{25},l_{52},l_{29},$ and $l_{92}$. Since there is no such ORN edge, the SOSG scheme overlays ORN node $v_{2}^{*}$ on top of ${\bar{n}}_{2}$ and uses the OEI scheme to insert these ORN edges $e_{23}^{*},e_{25}^{*},$ and $e_{29}^{*}$, as shown in Figure 11c. The newly matched NLM objects are removed from ${\bar{C}}_{N}$ and inserted to the matched NLM subgraph $G_{N}^{*}$. Finally, the key value of the last NLM node ${\bar{n}}_{1}$ is updated to zero ($k_{1}=0$).

Similarly, the last unmatched NLM node ${\bar{n}}_{1}$ in ${\bar{C}}_{N}$ has one ORN edge between ORN nodes $v_{1}$ and $v_{4}^{*}$. The SOSG scheme creates an ORN node $v_{1}^{*}$ at the projection point onto the extended ORN edge and inserts an ORN edge $\bar{v_{1}v_{1}^{*}}$ in Figure 11d. Finally, it also uses the OEI scheme to add the ORN edges $e_{12}^{*},e_{14}^{*},$ and $e_{110}^{*}$, which completely covers the unmatched NLM component ${\bar{C}}_{N}$.

### 5.4. Internal Structure Design of New ORN Node

**Figure 12:**
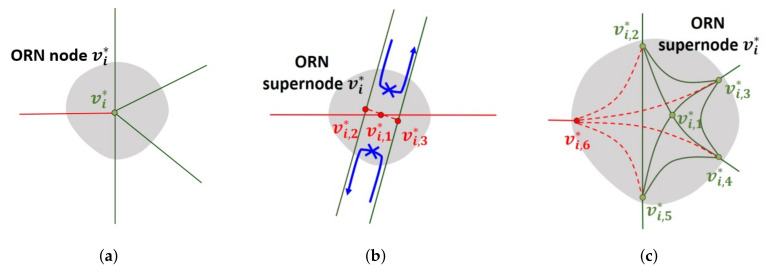
Addition of ORN subgraph to the existing ORN (super)node $v_{i}^{*}$ at (**a**) simple intersection, (**b**) dual carriage road, and (**c**) complex intersection.

Figure 12 shows a few examples of adding a set of new ORN edges to an existing ORN (super)node $v_{i}^{*}$, where green road objects represent the existing ORN subgraph, and red objects represent the new ORN subgraph by the SG scheme. There are three possible cases in the addition of a new ORN subgraph: (1) simple intersection, (2) dual carriage road, and (3) complex intersection.

To make the resulting ORN subgraph simple for the first two cases, our SG scheme restricts that *all ORN paths through the intersection must intersect at the same ORN node.* In addition, a new relation must be inserted into the ORN in order to reflect a turn restriction between a new ORN edge and an existing ORN edge. Since there is only one ORN node at a simple intersection, the new ORN edge is directly connected to ORN node $v_{i}^{*}$, as shown in Figure 12a. On the other hand, the ORN supernode $v_{i}^{*}$ for dual carriage road is placed in the middle of two parallel ORN edges. In Figure 12b, our SG scheme overlays an ORN node $v_{i,1}^{*}$ to this supernode and then requires that all additional ORN edges must intersect at this point. To interconnect the dual carriage edges to ORN node $v_{i,1}^{*}$, it also inserts two internal (red dashed) ORN edges, which connect this node and its projection onto two opposite ORN edges, i.e., ORN nodes $v_{i,2}^{*}$ and $v_{i,3}^{*}$. To avoid the u-turns via new internal ORN edges, it is also required to add an additional ORN relation that restricts the u-turns between two dual carriage edges.

However, it is not easy to define a single ORN node for connecting all ORN edges in a complex intersection due to the wide diversity of its internal structure. Figure 12c shows an example of ORN subgraph for complex intersection, where the set of ORN nodes are partitioned into two subsets: (1) the subset $V_{i,C}^{*}$ of *core* ORN nodes where each ORN edge is connected to another ORN node in the complex intersection, and (2) the subset $V_{i,B}^{*}$ of *boundary* ORN nodes having at least one ORN edge that connects to an ORN node outside the complex intersection. For example, $V_{i,C}^{*}=\left\{v_{i,1}^{*}\right\}$ and $V_{i,B}^{*}=\left\{v_{i,2}^{*},v_{i,3}^{*},v_{i,4}^{*},v_{i,5}^{*}\right\}$ in Figure 12c. In order to add a new ORN edge regardless of the internal structure, the SG scheme first adds a new boundary ORN node $v_{i,6}^{*}$ and then adds a new (red dashed) ORN edge that directly connects this new node with every other boundary ORN node. To reflect a turn restriction between a new ORN edge and an existing ORN edge, a new relation should be inserted into the ORN similarly to the previous two cases.

## 6. Numerical Results

In this section, we present the numerical results of the RNC between ORN and NLM at Yeoui-do island, Seoul, Korea: The former is extracted from the XML file at the official OSM website [45], and the latter is a shape file downloaded from the Korean ITS website [4]. Both road networks are imported to the PostgreSQL database for the RNC [46]. Table 4 shows the statistical information on the area, the number of nodes, the road segments, and the total length of road networks.

### 6.1. The Existing RNM Schemes

In this paper, the proposed AP scheme is compared with three existing node matching schemes, as follows:

- **Nearest first matching (NFM)**: In the NFM, the Euclidean distance between each NLM and ORN node pair that is within a distance threshold (100 m) is initially stored in a priority queue. At each step, the matching ($n_{i}^{*}$, $v_{j}^{*}$) with the smallest Euclidean distance in the priority queue is chosen, and then all remaining matchings with either NLM node $n_{i}^{*}$ or ORN node $v_{j}^{*}$ are removed from the priority queue.
- **Round-trip walk matching (RWM)** [28]: Given an initial matching, the RWM checks the topological consistency of the matching through the following three steps: First, it extracts the corresponding ORN node $v_{j}$ of each neighbor NLM node $n_{j}\in N\left(n_{i}\right)\backslash n_{i}$. Second, for each corresponding ORN node $v_{j}$, it examines the topological consistency by checking whether the corresponding ORN node $v_{i}$ of NLM node $n_{i}$ is also its neighbor ORN node or not. Finally, the ratio of the topologically inconsistent neighbor node is stored in a priority queue so that an NLM node with the highest topological consistency is extracted first for the final matching.
- **RWM with DBSCAN clustering (RWM-DC)**: Since both NFM and RWM are 1:1 node matching, they do not account for the LoD difference at a complex intersection. To mitigate this problem, the RWM-DC scheme combines the RWM with a clustering algorithm called the DBSCAN [40,42].

Given all pairs of matched NLM and ORN nodes, we use the score-based matching (SM) for the edge matching of the above three schemes [37]. The SM first computes a discrete similarity score based on multiple independent measures, i.e., the Hausdorff distance [39], orientation [31,39], mean perpendicular distance, and the nodal degree of endpoint nodes [28], and then chooses a pair with the highest score.

In our AP scheme, the threshold δ in Section 4.1 is chosen to the maximum width of the general highway and local road in Korea (34 m) [3].

### 6.2. RNM Results

In this section, we compare the RNM results of our AP scheme with those of three other RNM schemes. In Section 4.3, the matching result can be either CM, IM, PM, or MM. If we think of the RNM result as a binary classification, the CM can be interpreted as true positive, and the IM and PM can be interpreted as false positive. On the other hand, if we look at how a true ORN subgraph is matched to which NLM object, we can classify the matching result into three different cases, as follows: First, a matching scheme successfully finds the NLM object that corresponds to the true ORN subgraph, and the matching result becomes CM. Second, if it fails to find the right NLM object corresponding to the true ORN subgraph, the matching result is classified into *failed match (FM)*, which can be interpreted as false negative. Then, the FM can be further partitioned into PM, IM, and MM. Third, there is an exceptional case of binary classification, where the true ORN subgraph does not exist due to the errors in the OSM crowdsourcing process. Denoting the cardinality of the type-*m* matching result by |M(m)|, the precision, recall, and F1-score of the matching result can be defined as follows:

(4) $$Precision=\frac{\left|M(CM)\right|}{\left|M(CM)|+|M(IM)|+|M(PM)\right|},$$

(5) $$Recall=\frac{\left|M(CM)\right|}{\left|M(CM)|+|M(FM)\right|},$$

and

(6) $$F1\text{-}Score=\frac{2\times Precision\times Recall}{Precision+Recall},$$

respectively.

#### 6.2.1. Node Matching Results

**Figure 13:**
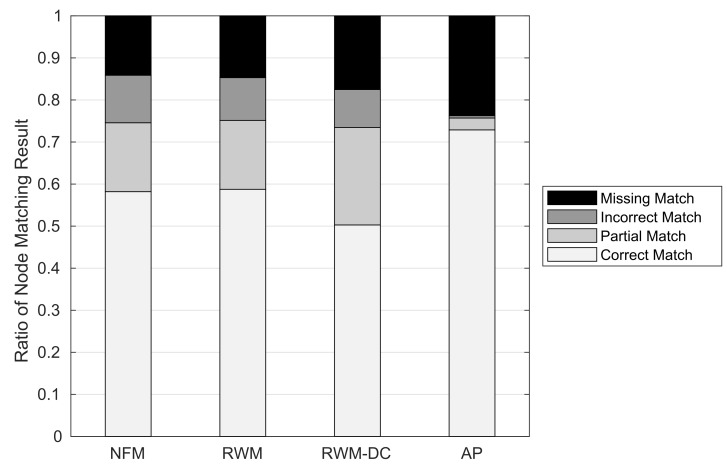
Ratio of node matching results.

Figure 13 shows the ratio of node matching results against the RNM schemes. We first observe that the proposed AP scheme can achieve an outstanding CM ratio of 0.73 at least 14.1% higher than the other RNM schemes. Its (CM, PM, IM, MM) ratio is (0.73, 0.028, 0.006, 0.237). The NFM and RWM schemes that do not support node clustering show almost similar RNM performance: The (CM, PM, IM, MM) ratios of NFM and RWM schemes are (0.582, 0.164, 0.113, 0.141) and (0.588, 0.164, 0.102, 0.147), respectively. The inaccurate node clustering of RWM-DC degrades the CM ratio to 0.503 while increasing the PM and MM ratios to 0.232 and 0.175, respectively. The excellent node clustering performance of the AP scheme originates from its low false positive ratio of 0.028, which is at least 8.29 times smaller than those of the other RNM schemes. Furthermore, the AP scheme has the lowest IM ratio of 0.006, while those of the other RNM schemes are at least 0.09. Since the node matching is performed sequentially for each NLM node, an IM of the previous NLM node may block the CM of a subsequent NLM node, which can significantly reduce the CM ratios of the other RNM schemes. The only problem with the AP scheme is its relatively high MM ratio, which will be addressed in Section 6.3.

**Figure 14:**
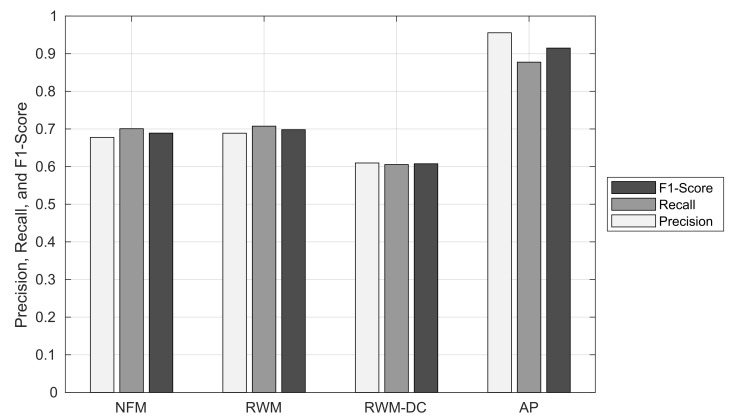
Precision, recall, and F1-score of node matching.

**Figure 15:**
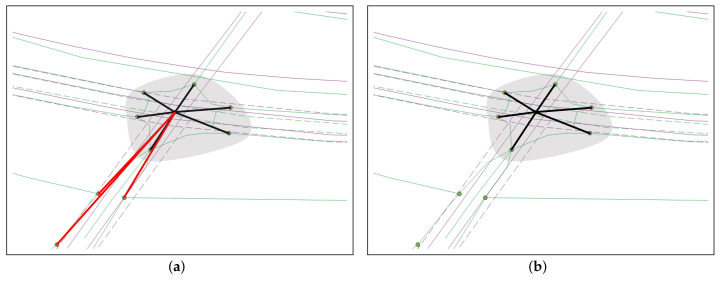
Example of node clustering in the (**a**) RWM-DG and (**b**) AP schemes.

Figure 14 shows the precision, recall, and F1-score of node matching in the NFM, RWM, RWM-DC, and AP schemes. It can be seen that the precision, recall, and F1-score of the AP scheme are at least 26.7%, 17.1%, and 21.7% higher than the other RNM schemes, respectively. Similar to the ratio of node matching result, the NFM and RWM schemes show a similar precision, recall, and F1-score: The difference in their performance is within 1.1%. The RWM scheme shows the lowest precision, recall, and F1-score due to its inaccurate node clustering. For example, the node clustering results of RWM-DC and AP schemes are shown in Figure 15a and Figure 15b, respectively, for the same complex intersection in the shaded region. While the AP scheme extracts the exact ORN nodes for the complex intersection, the RWM-DC scheme cannot distinguish three red ORN nodes belonging to minor intersections.

To summarize, the proposed AP scheme achieves an excellent node matching performance in terms of precision, recall, and F1-score compared with the existing three RNM schemes.

#### 6.2.2. Edge Matching Results

In this section, the edge matching performance of the AP scheme is compared with those of three existing RNM schemes in Section 6.1.

**Figure 16:**
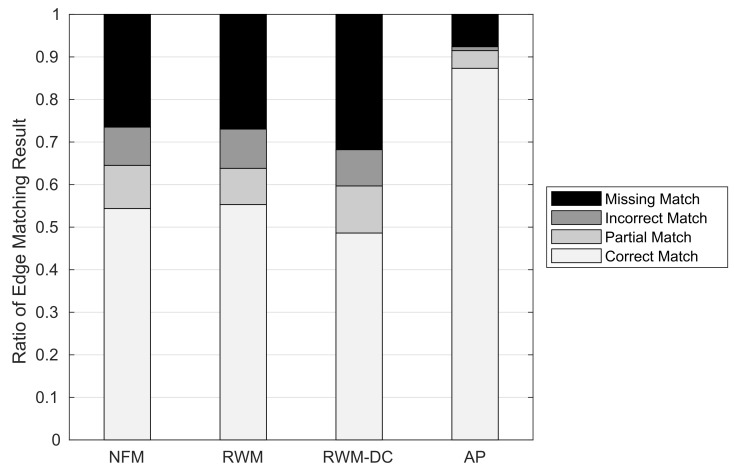
Ratio of edge matching results.

Figure 16 shows the ratio of edge matching results against the RNM schemes. We observe that the proposed AP scheme shows an excellent edge matching performance compared with the other RNM schemes. It has the highest CM ratio of 0.873 (at least 32% higher than the others), the lowest false positive ratio of 0.05 (at least 12.7% lower than the others), and the lowest MM ratio of 0.076 (at least 19.4% lower than the others). This outstanding performance of the AP scheme comes from its highly accurate node clustering at a complex intersection that minimizes both PM and IM ratios, which restricts the propagation of false positive in the subsequent edge matching. On the other hand, an inaccurate node matching of three RNM schemes results in a high MM ratio of edge matching. This is because in a generic road network with limited nodal degree, a change in the endpoints of ORN edge leads to a non-existent ORN edge with high probability.

**Figure 17:**
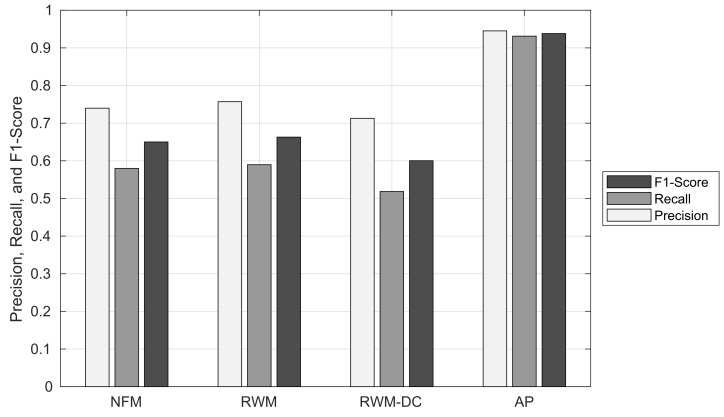
Precision, recall, and F1-score of edge matching.

Figure 17 shows the precision, recall, and F1-score of the edge matching against the RNM schemes. The AP scheme achieves superior edge matching performance with at least 18.8%, 34.1%, and 27.5% higher precision, recall, and F1-score, respectively, than the other RNM schemes. We also observe that the high MM ratio of three existing RNM schemes significantly degrades their recall performance.

From these results, we demonstrate that the proposed AP scheme can also achieve an outstanding edge matching performance compared with the existing RNM schemes.

### 6.3. RNC Results

In this section, we investigate how our APSG scheme can further improve the matching performance of the AP scheme. Table 5 lists the number of CM, PM, IM, and MM results of AP and APSG schemes at Yeoui-do island consisting of 177 NLM nodes and 434 NLM links. By adding ORN objects, the APSG scheme further improves the node matching performance of the AP scheme: The number of CM results is increased by 41, while the number of MM results is reduced by 42. As a result, it can increase the recall by 8.29% while slightly improving the precision by 0.49%. The APSG scheme also improves the edge matching performance compared with AP scheme: It improves both the precision and recall of AP scheme by 1.8% and 3.19%, respectively.

**Figure 18:**
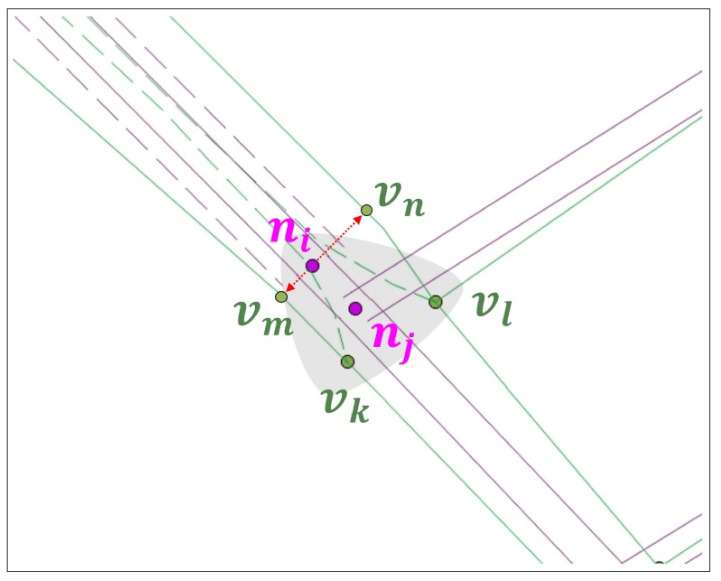
IM case in the APSG.

In Table 5, we also found the limitation of our APSG scheme in an exceptional node matching where an MM result of the AP scheme becomes an IM result by the APSG scheme. The shaded region in Figure 18 shows the complex intersection consisting of two nodes in both road networks. The NLM interprets this complex intersection as the combination of two intersections: $n_{i}$ connects a road with an underpass and $n_{j}$ connects three NLM links. On the other hand, the ORN interprets it as a single intersection with ORN nodes $v_{k}$ and $v_{l}$ interconnecting a dual carriage road, a road, and an underpass. This difference in the interpretation of road objects leads to 2:2 node matching, which cannot be addressed by our APSG scheme: In the AP scheme, the matching results for NLM nodes $n_{i}$ and $n_{j}$ are MM and PM, respectively. The APSG scheme projects NLM node $n_{i}$ onto the ORN nodes $v_{m}$ and $v_{n}$ in a dual carriage road, which changes the matching result to IM.

**Figure 19:**
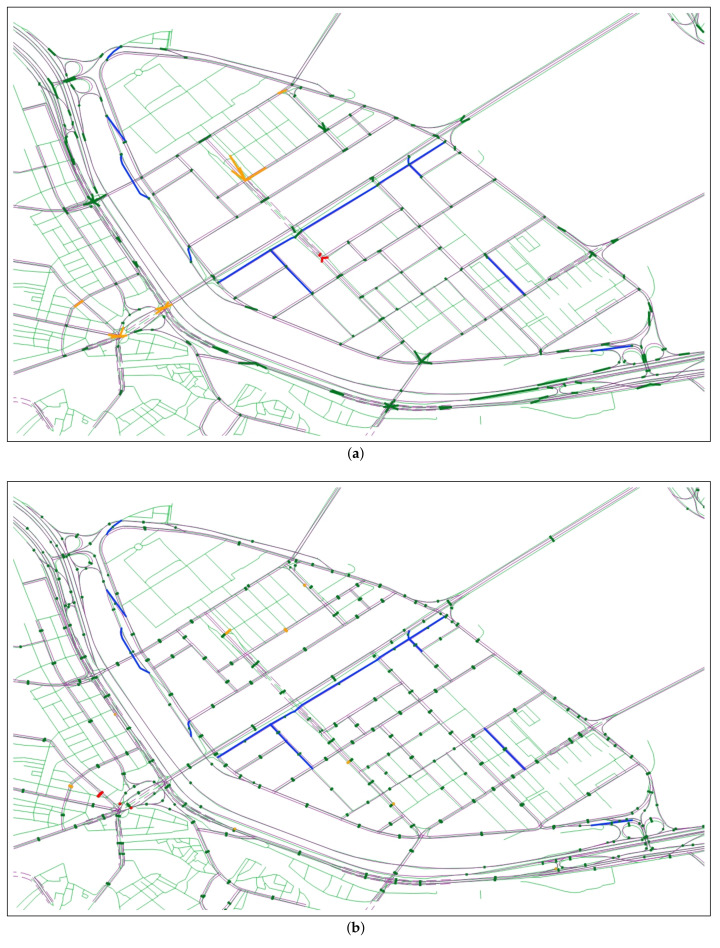
Matching results of APSG scheme: (**a**) Node matching and (**b**) edge matching.

Finally, the matching results of our APSG scheme at Yeoui-do island are shown in Figure 19, where Figure 19a,b illustrate the node and edge matching results, respectively. The blue subgraph represents the new subgraph added to the ORN by the APSG scheme. In addition, the thick dark green, orange, and red lines indicate the CM, PM, and IM results, respectively, between NLM and ORN objects. We can see that the proposed APSG scheme achieves outstanding node and edge matching performance.

## 7. Conclusions

This paper presents the APSG approach to the conflation between administrative and voluntary road networks. The AP scheme addresses the LoD problem of complex intersection through the partition of map area, extraction of candidate ORN subgraph, and aggregation to a supernode. For the unmatched NLM subgraph, the SG scheme sequentially inserts an ORN object while satisfying the connectivity with the matched NLM subgraph by the AP scheme. The numerical results show that our APSG scheme achieves an outstanding node and edge matching performance in terms of the precision, recall, and F1-score compared with the existing RNM schemes.

## Figures and Tables

**Table 1:** Characteristics of authoritative, proprietary, and voluntary road networks.

Characteristics	Authoritative [4]	Proprietary [5,6]	Voluntary [7]
Raw dataset	Accessible	Inaccessible	Accessible
Quality	Intermediate	High	Low
Level of detail	Low	High	High
Real-time data	Available	Available	Not available
Software packages	None	Limited	Abundant

**Table 2:** The *road_rank* attribute in the NLM.

*Road_Rank*	Explanation
101	Highway
102	Urban expressway
103	National road
104	Metropolitan city road
105	Aerial or inter-province road
106	Intra-province road
107	Intra-city or island road
108	Other roads

**Table 3:** Major *highway* tag of an OSM way.

*Highway* Tag Group	*Highway* Tag Value
Roads	*motorway*, *trunk*, *primary*, *secondary*, *tertiary*, *unclassified*, *residential*, *service*
Link roads	*motorway_link*, *trunk_link*, *primary_link*, *secondary_link*, *tertiary_link*
Special roads	*living_street*, *pedestrian*, *track*, *bus_guideway*, *escape*, *raceway*, *road*
Paths	*footway*, *bridleway*, *steps*, *path*, *cycleway*
Sidewalks	*sidewalk*
Cycleways	*cycleway*

**Table 4:** Statistical description of NLM and ORN in Yeouido.

Road Network	Spatial Extent	Number of Nodes	Number of Edges	Total Road Length
NLM	3.5 km × 2.8 km	177	434	124.74 km
ORN (Pruned)	3.5 km × 2.8 km	590	1005	140.67 km

**Table 5:** Number of matching results |M(·)| in AP and APSG schemes.

Number of	Node Matching					Edge Matching				
Matches	CM	PM	IM	MM	FM	CM	PM	IM	MM	FM
AP	129	5	1	42	18	379	18	4	33	28
APSG	170	5	2	0	7	418	12	4	0	16

**Figure A1:**
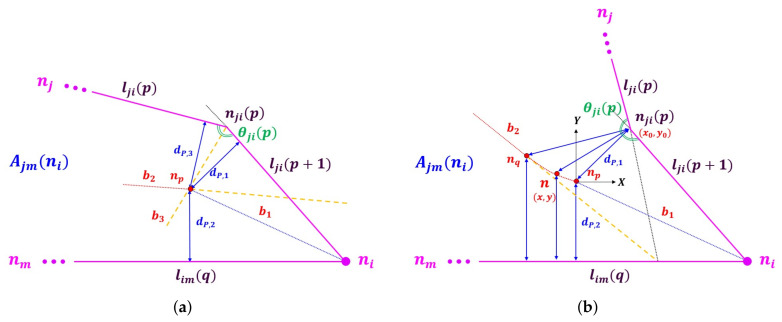
Construction of projection boundary in map area $A_{jm}\left(n_{i}\right)$ with (**a**) $\theta_{ji}\left(p\right)<180{}^{\circ}$ and (**b**) $\theta_{ji}\left(p\right)>180{}^{\circ}$.

## Data Availability

Publicly available datasets were analyzed in this study. This data can be found here: https://www.openstreetmap.org/ (accessed on 2 December 2021) and http://nodelink.its.go.kr/ (accessed on 2 December 2021).

## Appendix A. Transient Curve of Projection Boundary

In this appendix, we identify the transient curve of the projection boundary around a vertex in an intersection area. Figure A1 shows two examples of projection boundary in area $A_{jm}\left(n_{i}\right)$, where vertex $n_{ji}\left(p\right)$ connects two NLM line segments $l_{ji}\left(p\right)$ and $l_{ji}\left(p+1\right)$ in one projection side, and NLM line segment $l_{im}\left(q\right)$ is common on the other projection side. Starting from NLM node $n_{i}$, the projection boundary is the bisector $b_{1}$ of the angle created by $l_{ji}\left(p+1\right)$ and $l_{im}\left(q\right)$, and it is illustrated by the blue dotted line in both examples. It is clear that every point on this projection boundary should have the same projection distance to $l_{ji}$ and $l_{im}$, e.g., $d_{P,1}=d_{P,2}$. Our goal is to determine the point where the projection boundary deviates from $b_{1}$ and find the equidistant projection boundary between two NLM line segments $l_{ji}\left(p\right)$ and $l_{im}\left(q\right)$. Without loss of generality, we examine the projection boundary curve in two different cases: (1) The internal angle of vertex $n_{ji}\left(p\right)$ is less than 180° ($\theta_{ji}\left(p\right)<180{}^{\circ}$); and (2) It is greater than 180° ($\theta_{ji}\left(p\right)>180{}^{\circ}$).

Figure A1a shows an example where $\theta_{ji}\left(p\right)<180{}^{\circ}$. To find the point where the projection boundary deviates from $b_{1}$, we draw two additional bisectors that intersect with bisector $b_{1}$ at point $n_{p}$: bisector $b_{2}$ of the angle between $l_{ji}\left(p+1\right)$ and $l_{im}\left(q\right)$ and bisector $b_{3}$ of angle $\theta_{ji}\left(p\right)$. At point $n_{p}$, the projection distance to NLM line segments $l_{ji}\left(p\right)$, $l_{ji}\left(p+1\right)$, and $l_{im}\left(q\right)$ becomes the same. After point $n_{p}$, the projection boundary deviates from $b_{1}$ and becomes the red dotted line segment $b_{2}$.

When $\theta_{ji}\left(p\right)>180{}^{\circ}$, as shown in Figure A1b, bisector $b_{2}$ is similarly obtained from the crosspoint of $l_{im}\left(q\right)$ and the extended line of $l_{ji}\left(p\right)$. Next, we determine point $n_{q}$ on bisector $b_{2}$ so that its distance to point $n_{ji}\left(p\right)$ is equal to the projection distance to $l_{im}\left(q\right)$. It is clear that beyond point $n_{q}$, bisector $b_{2}$ becomes the projection boundary. The remaining problem is to determine the projection boundary between points $n_{p}$ and $n_{q}$. To address this problem, we first define a Cartesian coordinate whose *X*-axis crossing at the origin point $n_{p}$ is parallel to $l_{im}\left(q\right)$. We denote the Cartesian coordinate of point *n* on the transient boundary curve by (x,y). Similarly, the Cartesian coordinates of point $n_{ji}\left(p\right)$ are denoted by $\left(x_{0},y_{0}\right)$. Since y>0, the projection distance of point *n* to $l_{im}\left(q\right)$ becomes $y+d_{P,2}$ which must be equal to the distance between points *n* and $n_{ji}\left(p\right)$, i.e.,

(A1) $$\sqrt{{\left(x-x_{0}\right)}^{2}+{\left(y-y_{0}\right)}^{2}}=y+d_{P,2}.$$

Finally, the transient curve of projection boundary becomes a *parabola* satisfying the following equation:

(A2) $$y=\frac{{\left(x-x_{0}\right)}^{2}+y_{0}^{2}-d_{P,2}^{2}}{2(y_{0}+d_{P,2})}.$$
